# Descriptive norms and entrepreneurial intentions: the mediating role of anticipated inaction regret

**DOI:** 10.3389/fpsyg.2023.1203394

**Published:** 2024-01-31

**Authors:** Tae Jun Bae, Chong Kyoon Lee, Younggeun Lee, Alexander McKelvie, Woo Jin Lee

**Affiliations:** ^1^Hanyang University, Seoul, Republic of Korea; ^2^Hofstra University, Hempstede, NY, United States; ^3^James Madison University, Harrisonburg, VA, United States; ^4^California State University, Los Angeles, CA, United States; ^5^Syracuse University, Syracuse, NY, United States; ^6^Kookmin University, Seoul, Republic of Korea

**Keywords:** entrepreneurial intentions, theory of planned behavior, affect-as-information theory, emotion, cognition

## Abstract

Previous research has mainly focused on the cognitive-based theory of planned behavior (TPB) model to predict entrepreneurial intentions. However, given the close relationship between cognition and emotion, researchers may need to pay more attention to how emotional reactions help predict entrepreneurial intentions. To fill this gap, we apply both cognitive (i.e., descriptive norms) and emotional (i.e., anticipated inaction regret) aspects to understand predictors of entrepreneurial intentions. Specifically, we employ the affect-as-information perspective as a complementary theoretical lens to TPB to test whether the role of descriptive norms on entrepreneurial intentions is affected by anticipated inaction regret as a form of emotional reaction to descriptive social norms. We conducted two survey-based studies with diverse samples (i.e., online Mturk panels of adults in the US and undergraduate students in Korea). This study demonstrates (1) a positive and significant relationship between descriptive norms and entrepreneurial intentions and (2) a mediating role of anticipated inaction regret between descriptive norms and entrepreneurial intentions. Our results contribute to the entrepreneurial intentions literature by exploring the mechanism between cognition and emotion, and highlighting an indirect emotional link (i.e., anticipated inaction regret) in understanding entrepreneurial intentions.

## Introduction

Entrepreneurship has become a desirable career choice globally (Acs et al., 2008; Lewis et al., 2008). As a result, what makes people develop intentions to become entrepreneurs in their future careers has attracted scholarly attention (Souitaris et al., 2007; Liñán and Fayolle, 2015). The *theory of planned behavior* (TPB; Ajzen, 1991) has been a dominant theoretical framework for examining entrepreneurial intentions. According to meta-analyses, three variables of TPB (i.e., attitude toward the behavior, subjective norms, and perceived behavioral control) explain 38 percent to 62 percent of the intention variance across various contexts (Sheeran and Taylor, 1999; Armitage and Conner, 2001). Despite the theory’s explanatory power, some researchers across various contexts have argued for expanding the theory by including more predicting factors (Sheeran and Orbell, 1999).

In this regard, anecdotal evidence from real-world entrepreneurship cases demonstrates that people develop entrepreneurial intentions by observing what others do and experiencing an emotional reaction to such behaviors. For example, Mrs. Moon, who moved to Silicon Valley with her husband, told the media that she started a business because she felt left out in a place where everyone else, including student wives in Silicon Valley, attempted to start their own business. So, she finally started Bingle, which has a service called Viki for captioning videos worldwide. Unlike in the real world, little general research has been conducted on how other people’s entrepreneurial actions affect individuals’ emotions, ultimately influencing their entrepreneurial intentions.

Previous studies based on TPB have often operationalized subjective norms as *injunctive norms*, which is the perception of whether a behavior will be (dis) approved by important others. However, Ajzen and Fishbein (2005) note that subjective norms include *descriptive norms*, the perceptions of what significant others frequently do. That is, merely knowing what other people do (irrelevant of what others approve of) can influence one’s behavior by offering a criterion from which individuals are unwilling to deviate. Indeed, taking descriptive norms into account increased the explained variance in behavioral intention, over and above the three factors of TPB (Rivis and Sheeran, 2003; Smith-McLallen and Fishbein, 2008).

In addition, scholars claim that the cognitive-based TPB model may unnecessarily exclude the role of emotions when predicting behavioral intentions (Conner and Armitage, 1998; Treffers et al., 2017), which is potentially problematic as emotions and cognitions are inseparably intertwined (Piaget, 1981). Based on the *affect-as-information theory* (Schwarz and Clore, 1983), cognitive appraisal of a circumstance elicits powerful emotion (Ellsworth and Scherer, 2003), where emotions strongly influence people’s decisions and behaviors (Van der Pligt et al., 1997). Moreover, since entrepreneurship is future-oriented and people forecast their emotional reactions to future events (e.g., starting a business or not; Shepherd et al., 2009; Frederiks et al., 2019), we can expect that *anticipated inaction regret*, a negative self-blaming emotion stemming from inaction may act as an essential forecasted emotion in developing entrepreneurial intentions (Zeelenberg and Pieters, 2007; Zampetakis et al., 2015). Furthermore, studies such as Kahneman and Miller (1986) and Feldman and Albarracín (2017) show that anticipated regret over inaction manifests when a particular behavior is perceived as the norm. In other words, individuals would build the intention to become entrepreneurs because they do not want to regret not starting a business when it appears that everyone else has done so.

The notion above indicates that descriptive norms and anticipated regret of inaction are important additions to TPB to explain entrepreneurial intentions; however, these additional variables have not been systematically applied to entrepreneurial intentions over and above TPB’s predictors. Thus, this study aims to extend the literature by directly measuring and focusing on the role of descriptive norms in forming entrepreneurial intentions within the TPB framework. Furthermore, this study examines whether anticipated inaction regret mediates the descriptive norms-entrepreneurial intention relationship. To empirically test the proposed hypotheses, we employ two studies. Specifically, in Study 1, we recruited 222 participants through an online survey company in the United States, and in Study 2, we collected survey data from 128 undergraduate students at two private universities in Korea to test the direct impact of descriptive norms on entrepreneurial intention and the mediation impact of anticipated regret of intention on the relationship between descriptive norms and entrepreneurial intention.

We make several significant theoretical and practical contributions. First, while utilizing the theoretical lens of *affect-as-information theory*, this study extends our scholarly knowledge while exploring whether anticipated inaction regret acts as a mediator in the descriptive norms-entrepreneurial intention link. Many scholars claim that it is difficult to isolate the role of cognition and emotion (Lemerise and Arsenio, 2000), and how cognition and emotion differentially impact an individual’s intentions (Piaget, 1981; Izard, 1994). Additionally, a recent review of entrepreneurial intentions indicates that additional factors, beyond previously utilized cognitive factors, can enhance our comprehension of the intentions and behaviors of nascent entrepreneurs (Maheshwari et al., 2022). The extant literature has not fully addressed these factors (Hatak and Snellman, 2017; Neneh, 2019). While connecting cognition and emotion through the *affect-as-information theory*, we extend knowledge regarding the decisions and behaviors of nascent entrepreneurs and offer theoretical contributions based on this theorizing. Second, we advance the entrepreneurial intentions literature by highlighting the role of anticipated inaction regret. Although previous studies show the importance of descriptive norms on entrepreneurial intentions (Goethner et al., 2009; Meek et al., 2010), we explain the conditions under which these norms impact emotional factors as part of the descriptive norms-entrepreneurial intentions link. Third, this study provides a practical contribution for entrepreneurship educators and educational program developers by highlighting the importance of descriptive norms and the anticipated regret of inaction. In particular, emphasizing descriptive norms in entrepreneurship education via role models and connections with other entrepreneurs and acknowledging the feeling of regret can increase students’ entrepreneurial intentions.

The paper is structured as follows. We first discuss the literature related to entrepreneurial intentions. Then, we develop our arguments on the role of descriptive norms on entrepreneurial intentions and the mediating role of the anticipated regret of inaction, building on the affect-as-information theory. Next, we test our hypotheses using two distinct samples from the US and Korea. Finally, we conclude with discussions including contributions, limitations, and future research directions.

## Theory and hypothesis development

### Entrepreneurial intentions and theory of planned behavior

Entrepreneurial intentions are generally defined as the degree to which a person has formulated conscious plans to start a new business at some point in the future (Bird, 1988; Krueger et al., 2000). Understanding entrepreneurial intentions is important because it is believed that people with high entrepreneurial intentions are likely to become entrepreneurs (Krueger, 2009). Furthermore, in social psychology, intentions are “the most immediate and important predictors of behavioral performance” (Sheeran and Orbell, 1999, p. 2018). Similarly, various empirical studies found that entrepreneurial intentions are critical drivers for entrepreneurial actions (Kolvereid and Isaksen, 2006; Shirokova et al., 2016). For example, Schoon and Duckworth (2012) found that becoming an entrepreneur in mid-adulthood is significantly predicted by her entrepreneurial intention expressed during adolescence. Moreover, scholars empirically found a positive relationship between students’ entrepreneurial intentions and start-up activities (Van Gelderen et al., 2015; Shirokova et al., 2016).

To better predict entrepreneurial intentions, scholars have developed three distinct “intention-based models of entrepreneurship” – Implementing entrepreneurial ideas (IEI; Bird, 1988), Shapero entrepreneurial event (SEE; Shapero, 1975; Shapero and Sokol, 1982), and theory of planned behavior (TPB; Ajzen, 1991). Among the models, TPB has been a dominant theoretical framework for examining entrepreneurial intentions, using three predictors: (1) attitude toward the behavior, (2) subjective norms, and (3) perceived behavioral control (Schlaegel and Koenig, 2014; Kautonen et al., 2015). In a recent meta-analysis, the number of studies with TPB as a primary or a secondary theory was 68 out of 98 total studies, which shows that 70% of studies in the last 25 years have adopted TPB to examine intentions (Schlaegel and Koenig, 2014). This meta-analysis not only found that TPB has the largest predictability than other models but echoed that entrepreneurial intentions were based on attitude toward behavior (*r* = 0.43), subjective norms (*r* = 0.36) and perceived behavioral control (*r* = 0.56, *r* = 0.28; Schlaegel and Koenig, 2014). However, their results also reported that TPB determinants account only for 28% of the variance in entrepreneurial intentions, consistent with previous TPB meta-analyses that reported these variables cover 39% of the variance (Armitage and Conner, 2001). Accordingly, several researchers have argued further research is needed to increase the predictability of entrepreneurial intentions (Moriano et al., 2012; Hueso et al., 2021). To address this gap, we turn to the affect-as-information theory, which postulates that cognition and emotion influence behavioral intentions (Schwarz and Clore, 1983).

### Affect-as-information theory

According to affect-as-information theory (Schwarz and Clore, 1983), emotions reflect the underlying evaluation of a specific object (e.g., Ellsworth and Scherer, 2003), and people use their emotional states as a source of information for their judgments, decisions, and behaviors (Clore et al., 2001; Forgas and George, 2001). Emotional information is helpful in complex and uncertain contexts such as the entrepreneurial process (Baron, 2008). When people face an object, they ask themselves, ‘how do I feel about this situation?’ Then, they experience positive or negative emotions (Schwarz and Clore, 1983; Foo et al., 2009). In other words, cognitions are the primary emotional ingredients (Lazarus, 1991; Barsade and Gibson, 2007). Moreover, emotions provide compelling information about the personal value assigned to the object for the person experiencing these emotions (cf., Schwarz, 2001). In summary, this theory posits that the information regarding how one feels about an object can influence their reactions, such as intentions and behaviors toward this object (Schwarz and Clore, 1983). In a similar vein, an individual’s intention toward entrepreneurship does not entirely depend on cognitive components such as beliefs; emotions also play a role in influencing entrepreneurial decisions and behaviors (Cardon et al., 2005; Baron, 2008; Li, 2011; Welpe et al., 2012; Biraglia and Kadile, 2017; Ivanova et al., 2018). Scholars have argued that emotions shape cognition and that cognition shapes emotions because cognition is intertwined with emotions (Scherer et al., 2001; Robinson and Clore, 2002; DeSteno et al., 2004). Accordingly, affect-as-information theory can be an appropriate theoretical lens for exploring the role of cognitive and emotional factors and their interplay in entrepreneurial intention.

### Descriptive norms of entrepreneurship and the anticipated inaction regret

Descriptive norms are defined as the perception of others’ quantity and frequency of a behavior or what these individuals commonly do (Cialdini et al., 1990); it provides information about whether a behavior is typical/normal in a particular domain. By providing information about the typical behavior of others, descriptive norms set standards to analyze how far away people are from the norm of their social group about what is an accurate and effective behavior (Cialdini, 2012). Therefore, the degree of divergence from, or the convergence to, the descriptive norms can be the psychological gauge of people’s adaptability in their social groups (Schultz et al., 2007; Hornsey, 2008; Giannetti and Simonov, 2009). Therefore, engaging in behavior that conflicts with the descriptive norm may lead to negative affectivity among individuals, such as regrets. Indeed, when individuals perceive that other people typically engage in entrepreneurial activities but do not act similarly to their social references, they regret inaction. For example, Reb and Connolly (2010) demonstrated that information about prevalent behaviors generates the perception of social normality, making people consider socially abnormal choices emotionally regretful. Further, Feldman and Albarracín (2017) found that perceived prevalence for engaging in actions makes these actions standard, which leads to regret about the action not taken (termed inaction regret).

Descriptive norms may not only be related to how individuals feel about the action taken in the past but also to those about their future behaviors. For instance, individuals may start a business because they fear that they may regret not taking the chance, especially when other people typically do so. Such feeling is termed anticipated inaction regret. It is defined as a self-blaming emotional reaction resulting from an imagined outcome of what might happen to them if they do not act (Zeelenberg, 1999; Loewenstein and Lerner, 2003). Anticipated inaction regret may especially be salient when the norm is to take action—according to the norm theory, diverging from the set norm results in an intense feeling of regret because it is easier to imagine the alternatives to what actually happened as it is the normative decision (Kahneman and Tversky, 1982; Kahneman and Miller, 1986). Hence, when the norm is to take action and engage in entrepreneurial behaviors, people may easily anticipate regret about their inaction. Consistent with previous conceptualization and empirical findings, we expect that people who believe most others are entrepreneurs are more likely to perceive ‘being an entrepreneur’ as normal behavior. Then, they will anticipate intense regret if they decide not to be an entrepreneur as their career because they consider not becoming an entrepreneur as an atypical behavior. Accordingly, we argue that the appraisal of descriptive norms is a cognitive foundation likely to convey a higher level of anticipated inaction regret. Therefore, we propose the following:

*Hypothesis 1*: The stronger people’s descriptive norms of entrepreneurship are, the greater their anticipated inaction regret.

### Anticipated inaction regret and entrepreneurial intention

The affect-as-information theory has offered insights into the link between affect and intention (or behavior). When people perceive the discrepancy between actual and desired states, it produces negative emotions. Then, people are likely to regulate their behaviors or intentions to reduce these negative emotions (Carver and Scheier, 1990). Previous research has suggested that anticipated regret from future action or inaction will motivate people to engage (or not) in behaviors to prevent expected regret because people are regret-averse (Gilovich and Medvec, 1995; Reb, 2008). In particular, many empirical findings support that anticipated regret from not engaging in behavior increases people’s intention to act (Conner et al., 1999; Sheeran and Orbell, 1999; Armitage and Conner, 2001; Abraham and Sheeran, 2003).

In entrepreneurship, anecdotal evidence suggests that people are more likely to engage in entrepreneurship to avoid their anticipated regret if they do not pursue entrepreneurship. For example, Jeff Bezos, the founder of Amazon.com, frequently mentioned that he started his web-based bookstore to minimize his regret in the future (Brandt, 2011). He termed it a “regret minimization framework.” This claim has been supported by recent empirical evidence (Hatak and Snellman, 2017; Bouderbala, 2019; Neneh, 2019). Neneh (2019) found that anticipated inaction regret pushes individuals to act fast on their entrepreneurial intentions. Similarly, from two waves of survey data from the Finnish population, Hatak and Snellman (2017) found that anticipated regret from not engaging in business start-up activities is positively associated with undertaking nascent entrepreneurial activities. Bouderbala (2019) also analyzed the data from 266 students and showed that anticipated inaction regret directly and positively influences entrepreneurial intention. These empirical results imply that anticipated regret inaction is also significantly related to entrepreneurial intentions.

Based on the abovementioned reasoning, we propose that anticipated inaction regret by not becoming an entrepreneur is positively associated with a higher level of entrepreneurship intention to regulate the expected negative feelings. Thus, the following hypothesis is proposed:

*Hypothesis 2*: The stronger people’s anticipated inaction regret, the greater their entrepreneurial intentions.

### The mediating role of anticipated inaction regret

Affect-as-information theory posits that the information regarding how one feels about the object can influence the individual’s reactions such as intentions and behaviors toward the object (Schwarz and Clore, 1983). In particular, the preceding hypotheses are linked in an overall mediation model: Hypothesis 1 relates people’s descriptive norms of entrepreneurship to the level of anticipated inaction regret toward entrepreneurship, and Hypothesis 2 links anticipated inaction regret to the level of entrepreneurial intention. In entrepreneurship, extant research has demonstrated that descriptive norms play an important role in influencing entrepreneurial intentions. For example, people are likely to become entrepreneurs when their peers demonstrate entrepreneurial behaviors (Stuart and Ding, 2006; Nanda and Sørensen, 2010; Falck et al., 2012; Kacperczyk, 2013). Specifically, fund managers whose university peers have entered entrepreneurship are at a higher risk of entering entrepreneurship (Kacperczyk, 2013). Similarly, Nanda and Sørensen (2010) analyzed matched employer-employee panel data from Denmark and found that individuals’ propensity to become entrepreneurs is increased by workplace peers who have been entrepreneurs.

Our discussion implicitly suggests that descriptive norms may boost entrepreneurial intention by increasing *anticipated inaction regret*, referred to as the forward-looking emotional response of regret or upset from inaction (Armitage and Conner, 2001; Abraham and Sheeran, 2003). That is, an individual’s descriptive norms of entrepreneurship influence their level of anticipated inaction regret for not entering entrepreneurship, and the higher level of anticipated inaction regret converts the impact of the descriptive norms into implications for entrepreneurial intention. This mediation model is not intended to suggest that descriptive norms play no direct role in determining the level of entrepreneurial intention or that no other variables mediate the relationship between descriptive norms and entrepreneurial intention. This research, however, suggests that a combination of cognitive factors, such as descriptive norms, and emotional factors, such as anticipated regret of inaction, is necessary for a greater degree of entrepreneurial intention. In other words, based on affect-as-information theory, we argue that cognitive and affective factors influence an individual’s decisions and behaviors at the same time. Accordingly, our study argues that the role played by descriptive norms is more significant in the presence of affection from descriptive norms, especially the feeling of anticipated inaction regret. Therefore, we propose the following:

*Hypothesis 3*: Anticipated inaction regret mediates the relationship between descriptive norms and entrepreneurial intentions.

## Methods

We conducted two studies to test the hypotheses. By applying two studies, we attempt to circumvent common methodological concerns of single sample studies, by providing a replication using the same variables but in different contexts. The two-study approach helps provide more significant theory testing (Tsang and Kwan, 1999).

### Study 1

### Sample and data collection

The aim of Study 1 is to test the relationship between descriptive norms and entrepreneurial intentions with the mediating role of anticipated inaction. In 2015, we collected data from an Amazon’s Mechanical Turk (Mturk). Mturk is a crowd-based voluntary labor pool for online surveys in exchange for monetary incentives (Buhrmester et al., 2016). Mturk has been an increasingly popular tool for social science research because it is a valid recruitment tool and a fast and inexpensive way to collect a diverse sample of subjects (Berinsky et al., 2012; Minton et al., 2013). A total of 222 participants responded to a short online survey.^1^ All participants were not currently entrepreneurs.

### Measures

#### Dependent variable

##### Entrepreneurial intentions

Participants completed measures for entrepreneurial intentions with three items (Kolvereid, 1996; Souitaris et al., 2007): (1) “If you were to choose between running your own business and being employed by someone, what would you prefer?” (1 = Would prefer to be employed by someone; 7 = Would prefer to be self-employed), (2) “How likely is it that you will pursue a career as self-employed” (1 = Unlikely; 7 = Very likely), and (3) “How likely is it that you will pursue a career as employed in an organization?” (reverse-coded, 1 = Unlikely; 7 = Very likely). Items showed good internal reliability (*α* = 0.78).

#### Independent variable

##### Descriptive norms

We adopted two items from Sheeran and Orbell (1999). Respondents answered two questions of the following: “Of the people you know, what percentage of people are entrepreneurs?” rated on a 7-point scale ranging from 1 (0%) to 7 (100%), and “Of the five people you know best, how many are entrepreneurs?” rated on a 6-point scale ranging from 1 (none) to 6 (all; *α* = 0.75).

#### Mediator

##### Anticipated regret of inaction

We based our measure of anticipated regret of inaction on four items from previous research (Sheeran and Orbell, 1999; Abraham and Sheeran, 2003). Respondents were asked, “Would you regret it if you did not engage in entrepreneurship behavior (e.g., identify opportunities) in a few months [in a few years]?” and “Would you feel upset if you did not engage in entrepreneurship behavior (e.g., identify opportunities) in a few months [in a few years]?” The items used a Likert-type scale anchored at 1 = definitely no and 7 = definitely yes. The four items had a high reliability (*α* = 0.96).

#### TPB variables

In our analyses, we primarily controlled for TPB variables. *Attitude toward entrepreneurship* was measured by following the questionnaires validated in a previous study (Liñán and Chen, 2009). We also measured *subjective norms* and *perceived behavioral control* by following a study conducted by Kolvereid (1996).

#### Controls

We also controlled for participants’ age, gender, education level, entrepreneurial family background, entrepreneurial experience, and current occupation. *Age* was assessed by six different age groups (e.g., “1” = under 20s, “2” =20s – 30s, … “6” = over 60s). *Gender* was used by dichotomous variable of “1” = male, and “0” = female. *Education* was measured by three groups (e.g., “1” = less than a high school degree e, “2” = high school degree, “3” = college degree or higher). We also measured *entrepreneurial family background* and *entrepreneurial experience* by “1” = yes, and “0” = No. The current occupation was captured by four different groups (“1” = wage worker; “2” = temporarily unemployed; “3” = retired; “4” = students or others).

## Results

We conducted confirmatory factor analyses (CFAs) to examine whether the measures were appropriate for the latent constructs. We calculated parameter estimates using STATA 14 and the maximum likelihood method. The loading of each item was statistically significant (*p* < 0.001). To test whether the constructs were different from others, we tested the expected six-factor model, including the independent, dependent, mediator, and TPB variables. The results indicated a satisfactory fit to the data (*ꭓ^2^*[df] = 369.06[174], *p* < 0.01, SRMR = 0.071, CFI = 0.925, TLI = 0.91, and RMSEA = 0.071).

Table 1 provides mean, standard deviations, and correlations for all variables. We conducted ordinary least squares (OLS) regression to assess the direct relationship between descriptive norms and entrepreneurial intentions. Table 2 reports the results of regression models explaining entrepreneurial intentions. In Model 1 of Table 2, only control variables were entered. Level of education, entrepreneurial experience, and entrepreneurial family background were positively related to entrepreneurial intentions. This finding is consistent with previous empirical research that showed the positive effects of education (Davidsson and Honig, 2003), family background (Sheeran et al., 2005), and entrepreneurial experience (Mueller, 2006) on entrepreneurial entry.

**Table 1:** Descriptive statistics and correlations (Study 1).

Variables	M	S.D.	1	2	3	4	5	6	7	8	9	10	11	12	13
1. Entrepreneurial Intentions	3.9	1.61													
2. Age	3.1	1.19	−0.05												
3. Gender^a^	0.48	0.5	0.05	−0.04											
4. Education level	1.29	0.49	0.30^***^	−0.05	0.02										
5. Family Background^a^	0.18	0.38	0.17^**^	−0.22^**^	0.01	−0.06									
6. Entrepreneurial experience^a^	0.18	0.39	0.21^**^	0.08	0.16^*^	0.06	0.09								
7. Wage Employee^a^	0.75	0.43	−0.13^†^	0.01	0.20^**^	−0.27^***^	0.10	0.02							
8. Unemployed^a^	0.1	0.3	0.09	−0.07	−0.15^*^	0.16^*^	−0.04	−0.12^†^	−0.58^***^						
9. Retired^a^	0.05	0.21	0.11^†^	0.36^***^	−0.14^*^	0.08	−0.05	0.16^*^	−0.39^***^	−0.07					
10. Attitude	4.39	1.55	0.25^***^	−0.02	0.05	0.13^†^	0.07	0.02	−0.07	0.04	0.06				
11. Subjective norms	4.91	1.82	0.42^***^	−0.03	0.01	0.16^*^	0.11	−0.05	−0.01	−0.01	−0.01	0.06			
12. Perceived behavioral control	5.18	1.55	0.03	0.09	0.02	0.02	0.08	−0.04	−0.10	0.02	0.05	0.35^***^	0.06		
13. Descriptive norms	3.84	1.55	0.21^**^	−0.02	−0.09	0.03	0.39^***^	0.17^*^	0.12^†^	−0.07	0.05	−0.06	0.09	−0.03	
14. Anticipated Regret of Inaction	3.29	1.82	0.66^**^	−0.08	0.03	0.13^†^	0.17^***^	0.22^***^	−0.07	0.09	0.01	0.12^†^	0.27^***^	−0.00	0.27^***^

*N* = 222.

a Dummy variable.

† *p* < 0.10.

* *p* < 0.05.

** *p* < 0.01.

*** *p* < 0.001.

**Table 2:** Regression results of the effect of descriptive norms on entrepreneurial intentions (Study 1).

Variables	Entrepreneurial Intentions				
	Model 1	Model 2	Model 3	Model 4	Model 5
	Control only	TPB variables only	TPB variables w/controls	*Descriptive norms w/TPB variables*	*Descriptive norms w/TPB & controls*
Controls					
Age	−0.05		−0.05		−0.05
Gender^a^	0.03		0.02		0.03
Education	0.29^***^		0.20^**^		0.18^**^
Family Background^a^	0.17^*^		0.11^†^		0.06
Entrepreneurial Experience^a^			0.20^**^		0.20^**^
Occupation					
Wage Employee^a^	0.06		0.06		0.02
Unemployed^a^	0.12		0.13		0.11
Retired^a^	0.13		0.12		0.10
TPB variable					
Attitude		0.25^**^	0.20^**^	0.26^***^	0.21^**^
Subjective norms		0.17^***^	0.38^***^	0.39^***^	0.38^***^
Perceived behavioral control		−0.08	−0.07	−0.08	−0.06
Independent variable					
Descriptive Norms				0.18^*^	0.13^*^
*R^2^*	0.173	0.234	0.349	0.267	0.362
*F* value	5.57^***^	22.14^***^	10.24^***^	19.77^***^	9.87^***^
Mean VIF	1.702	1.097	1.572	1.078	1.582

*N* = 222; Standardized regression coefficients reported; VIF = variance inflation factor.

a Dummy variable.

† *p* < 0.10.

* *p* < 0.05.

** *p* < 0.01.

*** *p* < 0.001.

We entered TPB determinants only in Model 2 of Table 2. Attitude toward entrepreneurship (*β* = 0.17, *p* < 0.01) and subjective norms (*β* = 0.41, *p* < 0.001) were significant on entrepreneurial intentions. TPB variables accounted for 23.4% of the variance, consistent with previous research. As Hypothesis 1 predicts, we found a strong positive relationship between descriptive norms and entrepreneurial intentions. The results were shown in Models 4 and 5 of Table 3 (Model 3 of Table 3 includes all variables without the independent variable). Compared with Model 2, Model 4 showed that descriptive norms explained increments in *R*^2^ of 0.234 (in Model 2) to 0.267 after controlling for TPB variables. Descriptive norms significantly predicted entrepreneurial intentions over and above TPB determinants and contributed an additional 3.3% of the variance (*β* = 0.18, *p* < 0.05, in Model 4). In Model 5, the significant effect of descriptive norms on entrepreneurial intentions remains after controlling for all of the other variables (*β* = 0.13, *p* < 0.05, in Model 5). Model 5 illustrated that descriptive norms explained increments in *R*^2^ of 0.267 (in Model 4) to.362 after controlling for all controls and TPB variables. This finding provided support for Hypothesis 1.

**Table 3:** Results of mediation model (the PROCESS output), Study 1.

Variables		*M* (Anticipated Regret of Inaction)				*Y* (Entrepreneurial Intentions)		
		Coeff.	SE	*p*		Coeff.	SE	*p*
*X* (Descriptive norms)	*a*	0.23^**^	0.08	< 0.01	*c’*	0.03	0.05	0.59
*M* (Anticipated Regret of Inaction)		˗	˗	˗	*b*	0.46^***^	0.08	< 0.001
Age		−0.08	0.11	0.49		−0.03	0.07	0.67
Gender^a^		0.07	0.24	0.78		0.08	0.15	0.60
Education		0.14	0.25	0.59		0.55^***^	0.16	< 0.001
Family Background^a^		0.21	0.34	0.53		0.16	0.22	0.47
Entrepreneurial Experience^a^		0.99^**^	0.31	< 0.01		0.30^**^	0.21	0.15
Wage Employee^a^		−0.13	0.51	0.80		0.14	0.33	0.67
Unemployed^a^		0.63	0.58	0.28		0.31	0.38	0.42
Retired^a^		−0.18	0.77	0.81		0.82	0.50	0.10
Attitude		0.12	0.07	0.11		0.15^**^	0.05	< 0.01
Subjective norms		0.32^***^	0.08	< 0.001		0.28^**^	0.05	< 0.001
Perceived behavioral control		−0.06	0.10	0.51		−0.05	0.06	0.42
Constant		0.55	0.90	0.54		−0.40	0.58	0.49
		*R*^*2*^ = 0.2024 *F*(12, 209) = 4.4210, *p* < 0.001				*R*^*2*^ = 0.5757 *F*(13, 208) = 21.711, *p* < 0.001		
						CI		
		Effect		Boot SE		Boot LLCI	Boot ULCI	
Indirect effect of descriptive norms on entrepreneurial intentions through anticipated regret of inaction		0.1032		0.0449		0.0172	0.1922	

*N* = 222; The number of bootstrap samples for the bias-corrected interval is 5,000; CI = confidential interval.

aDummy variable.

**p < 0.01.

***p < 0.001.

We used Preacher and Hayes’s (2008) PROCESS macro to test the mediating effects of anticipated regret of inaction. This approach not only extends the Sobel test (Preacher and Hayes, 2004) but also allows us to test the indirect effect of the independent variable (i.e., descriptive norms) on the dependent variable (i.e., entrepreneurial intentions) through a mediator (i.e., anticipated regret of inaction). We utilized bootstrapping procedures with 5,000 resamples to place 95% corrected bootstrapped confidence intervals. The results from the bootstrapping analysis are in Table 4. In the first step, the proposed mediator, anticipated regret of inaction (*M*), was regressed on descriptive norms (*X*) to produce *a*. In the second step, entrepreneurial intentions (*Y*) were regressed on both anticipated regret of inaction (*M*) and descriptive norms (*X*), which yields *b* and *c’*, respectively. Results indicated that the indirect path from descriptive norms to entrepreneurial intentions through anticipated regret of inaction was statistically different from zero with 95% confidence, CI [0.0172, 0.1992]. This means that descriptive norms (*X*) lead to entrepreneurial intentions (*Y*) as a result of anticipated regret of inaction (*M*). Thus, hypothesis 2 was supported.

**Table 4:** Descriptive Statistics and Correlations (Study 2).

Variables	M	S.D.	1	2	3	4	5	6	7	8	9	10	11	12	13
1. Entrepreneurial Intentions (T2)	4.4	1.33													
2. Descriptive norms (T2)	4.41	2.0	0.22^*^												
3. Anticipated Inaction Regret (T2)	4.01	1.72	0.65^***^	0.38^**^											
4. Gender^a^	0.64	0.48	0.24^*^	0.02	0.10										
5. Entrepreneurial experience^a^	0.17	0.37	0.21^*^	0.23^*^	0.14	0.16									
6. Family Background^a^	0.37	0.48	0.13	0.47^***^	0.23^**^	0.04	0.01								
7. University^a^	0.63	0.48	0.34^***^	−0.07	0.16	0.14	0.17	0.01							
8. Entrepreneurship Class^a^	0.72	0.45	0.04	−0.07	0.13	−0.14	0.03	0.05	0.05^***^						
9. Attitude	5.44	1.24	0.68^***^	0.04	0.60^***^	0.13	0.12	0.12	0.25^**^	0.20^*^					
10. Perceived behavioral control	4.52	1.32	0.63^***^	0.16	0.67^***^	0.17	0.25^**^	0.17	0.25^**^	0.29^**^	0.71^***^				
11. Subjective norms	4.73	1.25	0.35^***^	0.16	0.36^***^	−0.03	0.03	0.29^**^	0.22^*^	−0.14	0.44^**^	0.40^***^			
12. Entrepreneurial Intentions (T1)	4.3	1.39	0.78^***^	0.25^**^	0.52^***^	0.26^**^	0.28^**^	0.21^*^	0.29^**^	0.06	0.59^***^	0.52^***^	0.32^**^		
13. Descriptive norms (T1)	4.31	1.83	0.26^**^	0.74^**^	0.38^**^	0.07	0.39^***^	0.34^**^	−0.06	0.05	0.16	0.27^**^	0.20^*^	0.39^***^	
14. Anticipated Inaction Regret (T1)	3.86	1.65	0.57^***^	0.24^**^	0.63^***^	0.06	0.17^***^	0.10	0.28^**^	0.08	0.44^***^	0.49^***^	0.23^*^	0.59^***^	0.39^***^

*N* = 128.

a Dummy variable.

* *p* < 0.05.

** *p* < 0.01.

*** *p* < 0.001.

### Study 2

In Study 2, we attempt to replicate the findings from Study 1, controlling for initial levels of all variables. However, we employ a sample from a different context. This offers distinct benefits. First, Study 2 provides a more externally valid test by using data from a different cultural context (i.e., Korea). This allows us to provide a replication test in a different geographic and cultural area, an important step for theoretical generalization (Tsang and Kwan, 1999). Second, we can rule out potential hysteresis effects by temporally separating our data collection. We do this by isolating and controlling for initial levels of our core variables reflecting predisposition factors from the initial level of intentions, norms, and anticipated inaction regret (Fayolle and Gailly, 2015). We collected data over two waves, consistent with previous research (Zhao et al., 2005; Souitaris et al., 2007). This research design over two waves enabled us to avoid common method bias (Podsakoff et al., 2003) and potential endogeneity problems (Menard, 2002).

### Sample and data collection

In 2019, we recruited undergraduate students from two private universities in Seoul, Korea. Students were enrolled in introductory management or entrepreneurship classes. Students who chose to participate in the survey were given extra credits. We used Brislin’s (1970) translation-back-translation approach to translate the questionnaires.

At Time 1, we sent an online survey to 296 students during the first 3 weeks of the semester at two universities. This resulted in 202 usable responses. Time 1 survey included questions on students’ demographics and measures of entrepreneurial intentions, descriptive norms, anticipated inaction regret, and the TPB variables. In Time 2, a follow-up online survey was emailed to 202 students who participated in the Time 1 survey. The Time 2 survey included the same variables as the first survey except for demographics. A total of 142 matched responses were received at Time 2. Due to incomplete and inconsistent information, 14 respondents were excluded, and 128 responses were used in the analyses (82 males, 46 females; 92 in entrepreneurship, 36 in management; age: *M*[*SD*] = 23.09 [2.03] years). The overall response rate was 63.4%. We found no significant differences between subjects lost through attrition and subjects remaining in the study in Time 2 concerning any of the captured background variables. Specifically, we compared subjects on several demographics, such as gender, entrepreneurial class, previous experience, and family background. From the chi-square test, gender (*χ*^2^[df] = 0.85[1], *p* = 0.36), entrepreneurship class (*χ*^2^[df] = 2.03 [1], *p* = 0.15), and family background (*χ*^2^[df] = 0.003[1], *p* = 0.96) of respondents and non-respondents in our survey were not significantly different from each other. Second, we compared group mean differences between respondents and 60 non-respondents on TPB variables and the independent variable (i.e., descriptive norms). An analysis of the variance of group means revealed no significant differences (see Appendix 1; *ps* > 0.05). We do not view response bias as notable in Study 2.

## Measures

### Dependent variable, independent variable, and mediator

The variables were measured at Times 1 and 2, using the identical items as in Study 1. The items showed good internal reliability for entrepreneurial intentions (*α*: T1 = 0.79, T2 = 0.79), descriptive norms (*α*: T1 = 0.81, T2 = 0.82), and anticipated inaction regret (*α*: T1 = 0.90, T2 = 0.91).

### TPB variables

In Study 2, we also controlled for TPB variables: attitude toward entrepreneurship (*α*: T1 = 0.85, T2 = 0.89), perceived behavioral control (*α*: T1 = 0.88, T2 = 0.91), and subjective social norms (*α*: T1 = 0.79, T2 = 0.80).

### Other controls

We also controlled for the following factors: participants’ *university* (1 = University A; 0 = University B), whether they were taking an *entrepreneurship class* (1 = yes; 0 = no), gender (1 = male; 0 = female), prior *entrepreneurial experience* (1 = yes; 0 = no), and *family background* (1 = entrepreneurial family; otherwise = 0).

To examine the discriminant validity of the constructs, we ran CFAs for Time 1 and Time 2 separately. We tested the expected six-factor model, including all independent, dependent, mediator, and TPB variables, as a one-factor model. The results indicated a satisfactory fit with the data in Time 1 (*ꭓ^2^*[*df*] = 299.57[188], *p* < 0.001, SRMR = 0.061, CFI = 0.94, TLI = 0.92, and RMSEA = 0.068). We found a similar pattern of satisfactory fit for Time 2 (*ꭓ^2^*[*df*] = 252.87[189], *p* < 0.001, SRMR = 0.052, CFI = 0.97, TLI = 0.96, and RMSEA = 0.053).

We conducted Harman’s one-factor analysis to check whether common method variance (CMV) influenced relationships due to the use of single-source data. The results showed that 39.36% at Time 1 and 45.07% at Time 2 of the total variance was explained, indicating that CMV was not a pervasive issue because it was below the ‘rule of thumb’ critical value of 0.5 (Podsakoff et al., 2003). We also examined the variance inflation factor (VIF) to check for multicollinearity. All VIFs were below the cutoff value of 10 (Gujarati and Porter, 2009), suggesting multicollinearity does not play a significant role in our results.

## Results

Table 4 provides the descriptive statistics and correlations for all of our variables. The results of the regression models for the relationship between descriptive norms and entrepreneurial intentions are presented in Table 5. In the first column of Table 5, we entered the control variables to explain entrepreneurial intentions. In the second column, we added the TPB variables (i.e., attitude toward entrepreneurship, perceived behavioral control, and subjective social norms). The TPB variables accounted for 32% of the variance. To test Hypothesis 1, we added our key independent variable (i.e., descriptive norms) in the third column of Table 5. We found that descriptive norms are positively and significantly related to entrepreneurial intentions (*β* = 0.20, *p* < 0.01). Descriptive norms explained an additional 2.7% of the variance beyond the TPB determinants and control variables. In the last column of Table 5, the significant relationship between descriptive norms and entrepreneurial intentions also remained after controlling for prior levels of entrepreneurial intentions and descriptive norms (*β* = 0.23, *p* < 0.01). Therefore, Hypothesis 1 is supported (Table 6).

**Table 5:** Regression results of the effect of descriptive norms on entrepreneurial intentions, Study 2.

Variables	Entrepreneurial Intentions			
	Model 1 Controls only	Model 2 Controls & TPB Variables	Model 3 Descriptive Norms w/Controls & TPB Variables	Model 4 Descriptive Norms w/Controls, TPB Variables, & Previous Levels
Controls				
Gender^a^	0.16	0.16	0.13^*^	0.05
Entrepreneurial Experience^a^	0.11	0.06^*^	0.10	−0.04
Family Background^a^	0.12	0.02	−0.07	−0.10^*^
University^a^	0.40^***^	0.09	0.14	0.05
Entrepreneurship Class^a^	0.22^**^	−0.09	−0.05	−0.06
TPB variables				
Attitude		0.40^***^	0.48^***^	0.27^***^
Perceived behavioral control		0.26^**^	0.22^**^	0.18^**^
Subjective norms		0.01	−0.01	−0.17^*^
Previous levels				
Entrepreneurial Intentions (T1)				0.56^***^
Descriptive Norms (T1)				−0.17^**^
Independent variable				
Descriptive Norms			0.20^**^	0.23^**^
*R^2^*	0.219	0.547	0.547	0.741
Δ*R^2^*		0.327^***^	0.027^***^	0.168^***^
*F* value	6.805^**^	17.797^***^	17.514^***^	29.988^***^
Mean VIF	1.164	1.164	1.728	1.951

*N* = 128; Standardized regression coefficients reported; VIF = variance inflation factor.

a Dummy variable.

* *p* < 0.05.

** *p* < 0.01.

*** *p* < 0.001.

**Table 6:** Results of mediation model (the PROCESS output), Study 2.

Variables		*M* (Anticipated regret of inaction)				*Y* (Entrepreneurial intentions)		
		Coeff.	SE	*p*		Coeff.	SE	*p*
*X* (Descriptive norms)	*a*	0.32^**^	0.07	< 0.001	*c’*	0.10^†^	0.05	0.06
*M* (Anticipated regret of inaction)		–	–	–	*b*	0.14^*^	0.06	< 0.05
Controls								
University^a^		−0.04	0.28	0.15		0.00	0.07	0.99
Entrepreneurial class^a^		−0.37	0.28	0.19		−0.18	0.19	0.35
Gender^a^		0.17	0.20	0.39		0.10	0.13	0.47
Entrepreneurial experience^a^		−0.50^†^	0.27	0.06		−0.05	0.18	0.80
Family background^a^		−0.11^*^	0.23	0.61		−0.25^†^	0.15	< 0.10
Attitude		0.31^**^	0.11	< 0.01		0.23^**^	0.08	< 0.01
Subjective norms		−0.00	0.09	0.96		−0.00	0.06	0.96
Perceived behavioral control		0.48^**^	0.11	0.57		0.13	0.08	0.11
Entrepreneurial intention (T1)		0.02	0.10	0.80		0.53^**^	0.08	< 0.001
Descriptive norms (T1)		−0.15^†^	0.08	0.06		−0.08	0.07	0.13
Anticipated regret (T1)		0.41^**^	0.08	< 0.001		0.01	0.06	0.88
Constant		−1.67^*^	0.54	< 0.01		−0.20	0.37	0.59
		*R*^*2*^ = 0.6671 *F*(12, 116) = 19.373, *p* < 0.001				*R*^*2*^ = 0.7705 *F*(13, 115) = 29.706, *p* < 0.001		
							CI	
		Effect	Boot SE		BootLLCI		BootULCI	
Indirect effect of descriptive norms on entrepreneurial intentions through anticipated regret of inaction		0.0454	0.0241		0.0057		0.0985	

*N* = 128; The number of bootstrap samples for the bias-corrected interval is 5,000; CI = confidential interval.

aDummy variable.

†*p* < 0.10.

*p < 0.05.

**p < 0.01.

**Figure 1:**
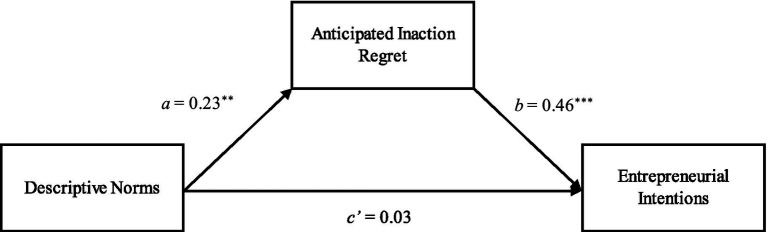
Results of mediation model in the form of a statistical diagram, Study 1 (^**^p < 0.01; ^***^p < 0.001).

**Figure 2:**
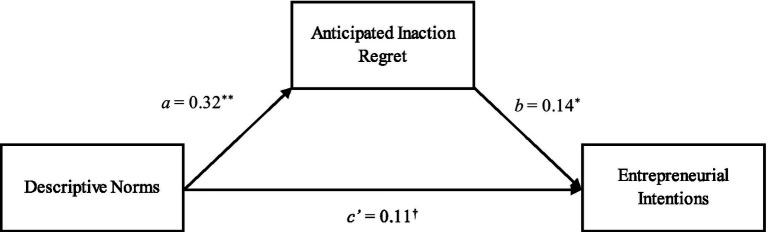
Results of mediation model in the form of a statistical diagram, Study 2. (^†^*p* < 0.10; ^*^p < 0.05; ^**^p < 0.01).

To examine the mediation effect of anticipated regret of inaction, similar to Study 1, we employed the PROCESS macro (Preacher and Hayes, 2008). The results illustrate that the indirect path (i.e., a product of a and b) between descriptive norms and entrepreneurial intentions through anticipated inaction regret was 0.045. The 95% CIs of the indirect path did not include 0 (lower bound = 0.0057, upper bound = 0.0985), thereby supporting Hypothesis 2. The mediating results are depicted in Figure 1 for Study 1 and Figure 2 for Study 2.

### Additional mediation analyses

To further eliminate the possibility of reverse causality in our mediation model, we tested additional alternative mediation models using descriptive norms and entrepreneurial intentions as mediators, respectively. First, one could argue that descriptive norms do not determine anticipated inaction regret. Instead, anticipated inaction regret may influence descriptive norms and then entrepreneurial intentions. In the presence of a high level of anticipated inaction regret, an individual may try more actively to take action (Ajzen and Sheikh, 2013) to minimize the emotion of regret by being much more sensitive to others’ behaviors, which more descriptive intentions will be perceived. In this case, anticipated inaction regret may boost descriptive norms, eventually increasing entrepreneurial intentions. To test this possible alternative explanation, we switched the mediator and independent variable (i.e., anticipated inaction regret → descriptive norms → entrepreneurial intentions). A 5,000-sample bootstrap analysis revealed that the 95% bias-corrected CI contains zero [−0.002, 0.115], suggesting a nonsignificant indirect effect. This empirically eliminates this as a viable alternative explanation.

Second, as Ajzen and Fishbein (1977) claimed that anticipated affect could follow inaction towards one’s intentions, entrepreneurial intentions may invoke anticipated inaction regret. To explore this possibility, we tested entrepreneurial intentions as a mediator between descriptive norms and anticipated inaction regret (i.e., descriptive norms → entrepreneurial intentions → anticipated inaction regret). Our analyses generated a 95% CI that excludes zero [0.005, 0.117], meaning that the alternative mediation was viable. We therefore further compared the proportion of variance of our original model and the reverse mediation model (Preacher and Kelley, 2011). When we assessed the ratio of indirect effects to total effects, the ratio of our original model was 0.31 compared to the alternative model having 0.14. This suggests that the point estimate of the proportion of the original mediation is higher. Therefore, this alternative explanation is inferior. Based on these alternative tests, we confirmed that our proposed mediating relationship (i.e., descriptive norms → anticipated inaction regret → entrepreneurial intentions) is valid and the strongest set of relationships among these key variables.

## Discussion

The results of this study indicate that the descriptive norms of entrepreneurship play a significant role in forming entrepreneurial intentions. Specifically, we found empirical support for Hypothesis 1 that descriptive norms are associated with entrepreneurial intentions. We also found support for Hypothesis 2, that anticipated inaction regret as an emotional factor mediates the link between descriptive norms and entrepreneurial intentions. In this paper, both hypotheses were supported by two studies employing diverse samples.

This research contributes to the literature on entrepreneurial intention by focusing on the emotional mechanisms behind entrepreneurial intention. Although scholars have recognized that emotion and cognition should be integrated (Lemerise and Arsenio, 2000), prior studies of entrepreneurial intentions have persisted with cognitive mechanisms (Maheshwari et al., 2022). However, recent studies highlighted the critical role of emotions as a determinant of the decisions and behaviors of nascent entrepreneurs (Hatak and Snellman, 2017; Neneh, 2019). Using the affect-as-information perspective (Schwarz and Clore, 1983), this research showed that descriptive norms exerted anticipated inaction regret as a future-oriented emotion and indicated that anticipated inaction regret has an informational function that could influence entrepreneurial intentions. This study thus supports the argument that emotion and cognition are simultaneously used for information processing but function differently; cognitions are a structure of information processing, and emotions are a facilitator for the process (Piaget, 1981; Izard, 1994).

This paper contributes to research on social influences on entrepreneurship (Liñán and Chen, 2009; Antončič and Auer Antončič, 2023). To date, exposure to others has impacted individuals’ entrepreneurial intentions (Nanda and Sørensen, 2010; Falck et al., 2012; Zapkau et al., 2015; Rahman et al., 2022). Previous studies on entrepreneurial intentions focused on injunctive norms, the perception of significant others’ approval or disapproval as a factor in enhancing one’s entrepreneurial intentions (see the review of Schlaegel and Koenig, 2014). This article not only explicitly distinguishes injunctive norms and descriptive norms (Cialdini et al., 1990) but also directly tests the descriptive norms and entrepreneurial intentions. Few studies have theoretically considered the descriptive norms of entrepreneurship on entrepreneurial intentions. Consistent with the argument that conformity to social references is rewarded with accuracy- or identity-based information (Giannetti and Simonov, 2009; Bar Nir et al., 2011), the results of two studies demonstrate a positive and significant relationship between descriptive norms and entrepreneurial intentions.

Furthermore, this paper added the emotional factor in forming entrepreneurial intentions. Despite the overt call to deepen our understanding of the role of emotions in entrepreneurship (Shepherd, 2015), empirical studies trying to assess the influence of anticipated inaction regret on entrepreneurial intentions are still largely absent in the literature. To the best of our knowledge, this study is the only study to investigate the anticipated inaction regret as a mediating mechanism to entrepreneurial intentions. This study thereby contributes to the literature on entrepreneurial intentions by developing a model capturing the additional predictive effects of descriptive norms and anticipated inaction regret that has not been extensively tested previously. This paper increases the predictability of individuals’ preferences for entrepreneurship, which answers the call for research on identifying various determinants of entrepreneurial intentions (Schlaegel and Koenig, 2014).

This research also has practical implications. First, for educators or program managers in entrepreneurship, this article suggests that descriptive norm-based pedagogy and materials can effectively form students’ entrepreneurial career intentions. Tian et al. (2022) and Ramadani et al. (2022) highlighted the role of entrepreneurial education on entrepreneurial intentions. Further, a recent study claims that entrepreneurial education should focus on developing social capital (Salamzadeh et al., 2022). Specifically, this study shows that by presenting entrepreneurship as a typical set of behaviors, descriptive norms can provide people with a time-and cost-saving shortcut for determining their entrepreneurial career intentions. Second, based on the finding that anticipated inaction regret plays a mediating role, educators or program managers should be aware that relying on descriptive norms alone may not be effective. Instead, it may be wise to support people in productively dealing with their anticipated inaction regret associated with an entrepreneurial career. This can be achieved by various tools such as reflections, role plays, or simulations (Shepherd, 2004), which suggest that emotional forecasting, especially anticipated inaction regret, is more critical for people to have higher entrepreneurial career intentions. Third, the results of this study show that people can be cognitively and emotionally biased toward being entrepreneurs when they believe that most are entrepreneurs among significant others regardless of their abilities or talents. Since the failure rate of new firms is significantly high (Wiklund et al., 2010), being motivated to be an entrepreneur without consideration of abilities is dangerous (Gompers et al., 2010). Therefore, people need to critically examine whether they are encouraged to be entrepreneurs because of their abilities or talents to create and manage new businesses so as not to be overly influenced by other people’s activities.

## Limitations and future research

This study is not without limitations. We believe that these offer opportunities for future research. First, we used self-reported data. Although all measures were previously validated, we acknowledge that using objective measures from various sources may be beneficial. Second, we cannot control for individual differences in cognition, such as perception, which may potentially moderate the relationship between descriptive norms and entrepreneurial intentions. For example, Rimal et al. (2005) show that the interaction of descriptive norms and perceived benefits from certain behaviors influences behavioral intentions. Thus, future research can examine whether the effect of descriptive norms on entrepreneurial intentions may vary based on how individuals perceive the benefits of entrepreneurship. Third, the results of this study are limited by the fact that this study focused on the breadth of descriptive norms via how much others engage in entrepreneurship rather than on the quality of the descriptive norms. Recent research has argued that prior entrepreneurial exposure is not unidimensional. For example, Zapkau et al. (2015) found that when people perceive their parents’ entrepreneurial activities as positive, they show a positive attitude toward entrepreneurship. We recommend future research to thoroughly delineate the heterogeneity of descriptive norms and their potentially varying influence on entrepreneurial intentions. Fourth, we exclusively focused on the anticipated negative feeling stemming from inaction. However, there are different paths through which anticipated emotions influence decisions, such as anticipated positive emotions (e.g., satisfaction) toward action (Fong and Wyer, 2003). Future work should clarify the relative impact of these different paths regarding anticipated emotions on entrepreneurial intentions. Fifth, there may be differences in factors influencing entrepreneurial intention between social and commercial entrepreneurship (Salamzadeh et al., 2013). Since we did not distinguish between social and commercial entrepreneurship, we hope future studies examine factors influencing entrepreneurial intention according to the type of entrepreneurship. Lastly, our empirical testing were based on two distinct contexts: the US and Korea. Even though the US and Korea have different cultures, this does not necessarily affect their entrepreneurial activities differently. According to GEM report (GEM, 2022), both countries have high levels of established business ownership and total early step entrepreneurial activity. Future research may evaluate the proposed model in various cultural contexts.

## Data availability statement

The raw data supporting the conclusions of this article will be made available by the authors, without undue reservation.

## Ethics statement

The studies involving human participants were reviewed and approved by the Institutional Review Board, Hofstra University. The patients/participants provided their written informed consent to participate in this study.

## Author contributions

All authors listed have made a substantial, direct, and intellectual contribution to the work and approved it for publication.

## Conflict of interest

The authors declare that the research was conducted in the absence of any commercial or financial relationships that could be construed as a potential conflict of interest.

## Publisher’s note

All claims expressed in this article are solely those of the authors and do not necessarily represent those of their affiliated organizations, or those of the publisher, the editors and the reviewers. Any product that may be evaluated in this article, or claim that may be made by its manufacturer, is not guaranteed or endorsed by the publisher.

## Footnotes

^1^Sample Characteristics: Gender (Male: 47.7%; Female: 52.3%), Occupation (Wage employee: 78.4%; Temporarily unemployed: 10.4%; Retried 5.0%; Student 6.3%), Age (under 20s: 1.4%; 20s-30s: 38.7%; 30s-40s: 28.4%; 40s-50s: 15.8%; 50s-60s: 11.2%; over 60s: 3.9%), Education (College and above: 72.1%; High school: 26.6%; less than high school: 1.4%).

## Appendix A.

Test of non-response bias: comparison with non-respondents, Study 2.

**Table:**

Variable	*df*	*F*	*p*-value
Entrepreneurial intentions at T1	1,200	1.929	0.17
Attitude at T1	1,195	1.322	0.25
Subjective norms at T1	1,195	0.825	0.37
Perceived behavioral control at T1	1,195	1.135	0.29
Descriptive norms at T1	1,198	0.141	0.71
Anticipated regret of inaction at T1	1,194	1.493	0.22
